# Introducing the chronic disease self-management program in Switzerland and other German-speaking countries: findings of a cross-border adaptation using a multiple-methods approach

**DOI:** 10.1186/s12913-015-1251-z

**Published:** 2015-12-28

**Authors:** Jörg Haslbeck, Sylvie Zanoni, Uwe Hartung, Margot Klein, Edith Gabriel, Manuela Eicher, Peter J. Schulz

**Affiliations:** Research Institute of the Kalaidos University of Applied Science Department of Health, Careum Research, Zurich, Switzerland; Institute of Communication and Health (ICH), Università della Svizzera Italiana, Lugano, Switzerland; Pflegezentrum Bachwiesen, Zurich, Switzerland; Careum Foundation, Zurich, Switzerland; Inselspital, Bern, Switzerland; University of Applied Sciences and Arts-Western Switzerland, School of Health Sciences, Fribourg, Switzerland

## Abstract

**Background:**

Stanford’s Chronic Disease Self-Management Program (CDSMP) stands out as having a large evidence-base and being broadly disseminated across various countries. To date, neither evidence nor practice exists of its systematic adaptation into a German-speaking context. The objective of this paper is to describe the systematic German adaptation and implementation process of the CDSMP (2010–2014), report the language-specific adaptation of Franco-Canadian CDSMP for the French-speaking part of Switzerland and report findings from the initial evaluation process.

**Methods:**

Multiple research methods were integrated to explore the perspective of workshop attendees, combining a longitudinal quantitative survey with self-report questionnaires, qualitative focus groups, and interviews. The evaluation process was conducted in for both the German and French adapted versions to gain insights into participants’ experiences in the program and to evaluate its impact. Perceived self-efficacy was measured using the German version of the Self-Efficacy for Managing Chronic Disease 6-Item Scale (SES6G).

**Results:**

Two hundred seventy eight people attending 35 workshops in Switzerland and Austria participated in the study. The study participants were receptive to the program content, peer-led approach and found principal methods useful, yet the structured approach did not address all their needs or expectations. Both short and long-term impact on self-efficacy were observed following the workshop participation (albeit with a minor decrease at 6-months). Participants reported positive impacts on aspects of coping and self-care, but limited effects on healthcare service utilization.

**Conclusions:**

Our findings suggest that the process for cross-border adaptation was effective, and that the CDSMP can successfully be implemented in diverse healthcare and community settings. The adapted CDSMP can be considered an asset for supporting self-management in both German-and French-speaking central European countries. It could have meaningful, wide-ranging implications for chronic illness care and primary prevention and potentially tertiary prevention of chronic disease. Further investigations are needed to tailor the program for better access to vulnerable and disadvantaged groups who might benefit the most, in terms of facilitating their health literacy in chronic illness.

## Background

The growing rates of chronic conditions pose major challenges for health care systems. A number of studies have been conducted to determine the effectiveness of self-management support systems, and programs have been developed to empower people living with chronic illness. Although findings from individual studies are mixed, the evidence largely suggests that supporting self-management has positive effects on individuals’ motivation, knowledge, and skills as well as improved quality of life, clinical outcomes, interactions with providers, and efficient use of health care resources [1–3].

One self-management intervention with a substantial evidence-base, the Chronic Disease Self-Management Program (CDSMP), was developed at Stanford University and has been broadly disseminated across populations and several countries [4–9]. The CDSMP comprises structured small-group interventions (2.5 h each) over six weeks and an accompanying reference book. The CDSMP workshop addresses people with a wide range of diseases, those with multiple morbidities, and their significant others and thus is one of the few self-management programs addressing those living with co-morbidities [10]. Besides the structured framework, a key feature is its peer-led approach. Individuals with a personal experience living with a chronic condition act as role models and are trained using a structured manual on how to lead workshops. The program includes aspects encountered throughout the chronic illness trajectory (e.g. fatigue, medication/symptom management, decision-making, communication with providers, and behavioural changes related to nutrition and exercise). The complex intervention also includes several active components: leaders encourage participants in goal setting and systematically support a group process with feedback and problem-solving activities [11–13]. The over-riding objective of the Stanford model is to enhance self-efficacy via goal setting and action planning, leading to improved self-management behaviours with positive effects on quality of life and health-related outcomes [14].

The effectiveness of the CDSMP has been demonstrated in studies across age groups and diverse cultural and ethnic backgrounds. The majority of participants have been women (>75 %) between 50–65 years of age living with a chronic condition [6]. Studies have identified the most positive outcomes among a middle-aged population [15]. Outcome measures have usually focused on self-efficacy, the program’s conceptual cornerstone, which was often significantly increased up to six months after the end of the program, even though it may decline later [4, 5, 16–18]. The CDSMP also can lead to significant improvements in health behaviours, including increased exercise, and enhanced cognitive symptom management and communication with healthcare providers [5, 18, 19]. Significant positive changes in health indicators like self-rated health, disability, fatigue, quality of life, and health distress have been identified [5, 16, 19–21]. Moreover, several studies detected statistically-significant differences in health services utilisation, like emergency room visits and hospitalizations [19, 21–23]. Data also suggest the CDSMP may reduce healthcare costs [24]. Subjectively, the workshops are well-received and patients report feeling more knowledgeable and inspired to better manage their condition [7, 10, 25]. Moreover, the potential to inspire and encourage individuals to take further action is evidenced by individuals subsequently joining self-help groups or participating in volunteer work [25, 26]. In addition to peer leader role-modelling, the group process is highly valued because it can facilitate social connections, reduces isolation, improve coping skills, and may help participants to accept their situation [7, 25, 27–29].

Critiques of the CDSMP also include the fact that some studies have not identified changes in perceived self-efficacy or in health [10, 30], a finding that may be due in part to high levels of self-efficacy at baseline. Other studies failed to detect positive effects on health status [17], health services utilisation at six months follow-up [5, 16], or total health care expenditures [17]. Participants have voiced challenges too. These include concerns related to cultural adaptation, potential language barriers, the program’s restrictive timeframe, and the workshop itself being overly strenuous and requiring considerable resources [8, 10, 31]. Along with a number of methodological challenges and analytical issues related to this program [32], some consider the benefits as relatively modest, and there is an ongoing call for more rigorous research to strengthen the evidence base for the CDSMP [33–35]. Indeed, the international dissemination of the model and its impact have been critically assessed [32, 36, 37]. Despite successes, there are doubts that the program can reach those most likely to benefit; e. g., vulnerable populations with limited health literacy; that group dynamics could also have negative impact on the participants due to social comparisons; that the underlying ethos of an activated expert patient and ‘good’ self-manager may trigger inequalities; that the CDSMP’s psychological assumptions as well as fixation on self-efficacy as an outcome instead of a mediator may be misleading and lead to a marginalization of its other, equally important theoretical elements like chronic illness work identified by Corbin and Strauss [38]. Others have criticized that its emphasis on peer-led self-management support may neglect the value and need of support provided by healthcare professionals [25, 39–41].

Given the existing evidence on the CDSMP and the range of its benefits and challenges, recently-published findings reinforce the program’s apparent association with medium-term improvements in self-efficacy, health status, and health care utilization. As observed changes may persist long-term, the CDSMP can be considered a valuable contribution to comprehensive chronic care and public health strategies [13, 42, 43]. However, to our knowledge, no evidence exists to date supporting the systematic adaptation and implementation of the CDSMP in French-and German-speaking countries in central Europe. We aimed to perform a systematic adaptation and implementation process. This paper presents findings from an evaluation of this implementation process examining whether the CDSMP could be effective in Austria, Germany and Switzerland. In addition, we set out to determine the cultural acceptability and utility in German-and French-speaking European countries as a useful supplement to existing chronic care strategies.

## Methods

### Systematic adaptation and implementation process

To date, relatively few studies have shed further light on how the Stanford model, as a complex self-management intervention, can be adapted not only to specific populations or ethnic groups, but also across borders into other language regions [44–47]. Some of these papers were published after the design and initiation of the project at hand and very few recommendations were made about how to structure the systematic cross-border adaptation and adoption of a self-management intervention.

**Fig. 1:**
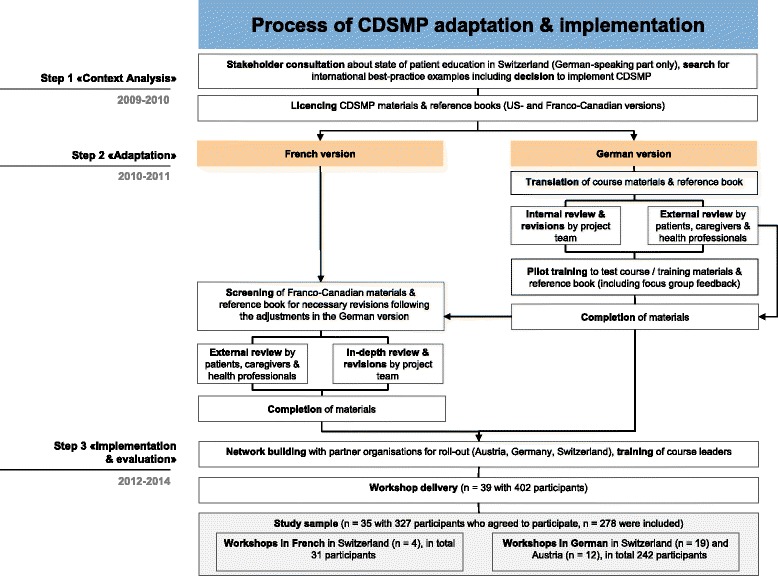
Process of cross-border adaptation and adoption of the CDSMP in Switzerland and German-speaking Europe

In 2009, a national and international context analysis (step 1) of present patient education approaches was conducted by Careum, a non-profit foundation based in Zurich, Switzerland [48]. It was the initial stepping stone for a three-step systematic process (see Fig. 1) for adapting and implementing the CDSMP. One analytic outcome was identifying a direct need as there was a paucity of evidence-based self-management support and patient-centred peer-led programs in Switzerland.

Step 2 was to create a German version of the Stanford model (Fig. 1). Other German speaking stakeholders in Austria and Germany were engaged for the adaptation process and subsequent pilot implementation. Patient engagement was considered central to the process, and close collaboration with healthcare professionals, scholars, international experts experienced with the Stanford program, and various organisations in the healthcare setting ensured that Stanford’s CDSMP manuals and reference books were adequately adapted and implemented into the program «Living healthy and actively» (German acronym: Evivo)^1^

In 2010, all English materials (including the reference book) were translated into German. Then sections were reviewed by external patients, caregivers and healthcare professionals. Feedback was collected via structured questionnaires and workshops aimed at culturally tailoring the content for linguistic appeal enabling program delivery to a German speaking population. This included adapting semantics and metaphors that were not self-explanatory for the European German-speaking population. Specifically, marginal text lines were introduced as structural aids in the German version of the reference book [49] making it more reader-friendly, enabling skim reading, and highlighting key messages. Both the book and course content on nutrition were re-written in collaboration with the Swiss Society for Nutrition. Content on physical activity, endurance and strength was reviewed by the Swiss Rheumatism League and adapted using figures as visual aids to illustrate physical activities making the material more appealing and user-friendly. Based on prior work conducted in the United Kingdom on advanced care planning [31], this topic was excluded from German version of the reference book. Country-specific booklets were created providing an annotated bibliography and listing of local support organisations.

Pilot training sessions were used to pre-test the materials facilitating revision of certain program activities. For example, the 2006 Stanford version includes one brainstorming activity to address participants’ «problems» with healthcare professionals and the healthcare system. From the patient and caregiver points of view, this was perceived as too deficit-oriented, so the broader term «experience» (German translation: Erfahrung) was introduced. These amendments were approved by Stanford as well as by international experts familiar with the program.

Such revisions also helped guide the adaptation of the Franco-Canadian CDSMP materials for the French-speaking Swiss population (Fig. 1). Relevant sections in the manuals, reference book [50] and service booklet were checked via an internal and external review and, where necessary, were either translated or changed. Again, participating patients, caregivers and experts pointed out changes relevant to the French-speaking part of Switzerland making the adapted program appealing to residents of this linguistic region of the country.

In step 3 (2011–2012), trainings were held for leaders from partner organisations in preparation for program launch. This was initially started in German and subsequently in French across diverse settings (health insurance, home care, community services, hospitals and primary care centers). Since our objective was to determine the CDSMP’s acceptance in Switzerland, there was no intention to address subgroups of specific chronic diseases. However, during implementation, some partner organisations implemented workshops specifically for HIV-positive individuals and members of ethnic minorities. In line with the open innovation character of the project and to address expressed needs and expectations, workshops in German were offered, as is, to these subgroups, if enough potential participants were enrolled.

### Design

A multiple-methods design was employed to explore the perspectives of participants attending Stanford’s CDSMP in Austria, Germany and Switzerland. We combined a longitudinal structured survey with qualitative methods (focus groups and interviews in both German and French). The 3-part survey was comprised of self-reported questionnaires at the beginning and end of each small group workshop respectively (wave 1, wave 2) and a final follow-up assessment (4–6 months post-workshop completion, wave 3). After the workshop (approximately 1 week) a subset of survey respondents participated in the qualitative focus groups to gain additional insights on their experiences. Data were collected on the geographical/language region (e.g., via home healthcare or community-based delivery in Switzerland or Austria) and how they assessed its impact. In addition, individual interviews were used to explore the experiences of the aforementioned subgroup of female immigrants. The study was approved by the Internal Review Board of the Department of Health for Kalaidos University of Applied Science of Switzerland, in Zurich, Switzerland and study participants provided written informed consent.

### Sampling and recruitment

Between January 2012 and May 2014 we recruited a convenience sample from 402 total participants attending 39 small-group CDSMP workshops across German and French-speaking Switzerland and Austria (German-only workshops). No workshops were conducted in Germany due to recruitment challenges. Thus, no data from Germany are reported. The lack of workshop participants in Germany was unanticipated and may have resulted from the lack of a coordinated roll-out plan there (see endnote 2 on the more successful later-on developments in Germany).^2^Workshops were publicized via media announcements and reports, newsletters, mailings and leaflets, and by direct invitation from workshop staff. Participants were adults, able to attend the 6-week CDSMP course who were willing to participate in the evaluation and able to communicate adequately (German or French respectively).

### Instruments

#### Survey phase

Data were collected using self-report questionnaires (German or French respectively). The questionnaires included questions on socio-demographic characteristics. In addition, information was collected on participants’ perceptions of the workshop experience and organisation, course materials (a reference book), as well as perceived changes in health status, health behaviour and patient-provider-interactions. The measures assessing course experience, self-management skills, and lifestyle behaviours utilized 7 and 10 point Likert-type scales respectively. Questions were developed for this study based on the current published CDSMP literature.

Perceived self-efficacy was measured using the validated German version of the Self-Efficacy for Managing Chronic Disease 6-Item Scale, SES6G [51]. Questions are scored on a 10-point scale with higher cumulative scores reflecting higher levels of self-efficacy. For this study, the SES6G questions were translated into French, and then translated back into German to ensure an accurate French translation that was subsequently used for the pilot study.

#### Qualitative phase

A semi-structured interview guide was developed for focus groups and interviews (Table 1). Open-ended questions explored participant expectations of the workshop and their appraisal of the significance of content, topics and the reference book. Specific questions evaluated the workshop leaders and the group process, as well as any changes in their social context and everyday life after workshop participation.

**Table 1:** Topics for interviews and the focus group guide

Topic	Open-ended questions related to
Expectations	• hearing about the course and from whom
	• expectations people had before the course
Course content and process	• what participants liked/disliked
	• whether they had learned something new
	• topics they have missed or were affected by
	• structure and procedures of the program
	• group experience and social connectivity
	• expectations and experiences
	• appraisal of leaders and workshop delivery
After the course	• changes in everyday life, with specific examples
	• positive, challenging and/or unexpected changes
	• responses of significant others
	• subjective indicators of successful outcomes
	• contemplations about becoming a peer leader
Reference book	• experiences and expectations related to the material
	• its usability and layout
	• room for improvement

### Data collection and analysis

Survey data were collected when a particular workshop started and ended with a self-administered questionnaire that the participants received on-site (waves 1 and 2), and then by mail 4–6 months later (wave 3). Each questionnaire was coded to protect participant identity. In the qualitative phase of the study, the focus groups and interviews typically occurred at the local workshop site and sessions were audio-taped and field notes and memos were recorded by the research team during encounters. Recordings were transcribed and participant identifiers removed.

Quantitative data are reported using descriptive statistics (SPSS Version 21). Qualitative data underwent thematic analysis based upon the principles of Grounded Theory, in particular constant comparative analysis combined with a coding approach to identify emerging codes and synthesise them into categories [52, 53]. Preliminary findings were re-examined using peer discussions within the research team and an interdisciplinary group of healthcare researchers to assure the trustworthiness of results.

## Results

### Sample

Of the 402 participants in 39 small group workshops (2012–2014) 327 (attending 35 workshops) agreed to participate. In total, 278 (85.0 %) were eligible for inclusion in the study (Table 2). The majority of workshops were delivered in the German-speaking parts of Switzerland (*n* = 19) and Austria (*n* = 12), with four workshops involving a total of 31 participants conducted in French. Of the 278 total participants, 56 also were involved in the qualitative phase (Table 3). Eight focus groups were conducted to examine participants’ experiences across healthcare settings in Austria and Switzerland. Pre-workshop questionnaires (wave 1) were completed by 278 participants and by 250 participants in wave 2. In the follow-up period (4–6 months post-workshop, wave 3), 138 participants returned the questionnaire. The vast majority (>90 %) of the 278 were middle-aged women living with a variety of chronic conditions. Approximately 80 % of participants attended because they were chronically ill, about one third because they had a friend or family member with a chronic condition, and only about 10 % had a spouse or partner who was chronically ill (multiple answers possible). From the single workshop delivered to female immigrants, nine participated in a follow-up focus group and their data were included in the analysis. Overall, two out of three of the study participants (66 %, *n* = 184) attended all workshop sessions, and one fourth (24.2 %, *n* = 79) missed one session only. Due to the small sample size and heterogeneous workshop settings, no meaningful patterns emerged between countries or language areas, in terms of differences in workshop attendance.

**Table 2:** Survey participant characteristics

Characteristics	Participants at the start of workshop (*n* = 278)
Average age	58.3 yrs (20–87)
Sex^a^	
Female	242 (88.3 %)
Male	32 (11.7 %)
Family status	
Married	124 (44.6 %)
Cohabitating	22 (7.9 %)
Divorced	45 (16.2 %)
Widowed	29 (10.4 %)
Single	48 (17.3 %)
Partnership	6 (2.2 %)
Not specified	4 (1.4 %)
Employment^b^	
Unskilled worker	16 (5.9 %)
Trained employee	101 (37.5 %)
Freelance/self-employed	34 (12.6 %)
Apprenticeship	10 (3.7 %)
Retired	136 (50.6 %)
Incapacitated for work	32 (11.9 %)
House wife/husband	77 (28.6 %)
Health^b^	
Chronic back pain	97 (35.1 %)
Arthritis	80 (29.0 %)
Other musculoskeletal disease	42 (15.2 %)
Mental health disease	59 (21.4 %)
Diabetes	39 (14.2 %)
Cardiovascular disease	25 (9.1 %)
Living with chronic conditions^b^	
Chronically ill	241 (87.0 %)
Significant other, friend	84 (30.3 %)
Health professional	60 (21.7 %)
Spouse living with chronic condition	38 (13.7 %)

^a^ *n* = 274, missing data; ^b^multiple answers possible

**Table 3:** Focus group and interview participant characteristics

Focus group FG, participants	FG 1 (*n* = 5)	FG 2 (*n* = 9)	FG 3 (*n* = 5)	FG 4 (*n* = 9)	FG 5 (*n* = 7)	FG 6 (*n* = 9)	FG 7 (*n* = 3)	FG 8 (*n* = 6)	Interview 1	Interview 2	Interview 3	Sum (n)
Significant other	1	-	-	1	2	-	-	4	-	1	-	9
Sex												
Female	3	5	1	9	7	9	3	6	1	1	1	46
Male	2	4	4	-	-	-	-	-	-	-	-	10
Age Ø	60 (54–66)	56 (43–71)	54 (43–65)	66 (33–81)	64 (56–82)	58 (39–74)	52 (42–64)	51 (46–57)	51	-	47	
30-49 years	-	3	2	1	-	2	1	3	-	-	1	13
50-69 years	2	5	3	5	6	2	2	2	1	-	-	28
70-89 years	-	1	-	3	1	3	-	-	-	-	-	8
Not specified	3	-	-	-	-	2	-	1	-	1	-	7
Education												
Secondary school	-	2	-	1	1	5	-	2	-	-	-	11
University degree	-	4	5	1	3	1	-	2	-	-	-	16
Apprenticeship	-	-	-	6	2	1	3	1	1	-	-	14
Not specified	5	-	-	-	-	2	-	1	-	1	1	10
Family status												
Living alone	3	3	2	5	4	2	2	1	-	-	-	22
Living with partner	-	1	3	1	1	3	1	-	1	1	-	12
Living with family	2	3	-	1	1	2	-	5	-	-	1	15
Not specified	-	2	-	2	1	2	-	-	-	-	-	7
Chronic conditions												
Heart disease	-	1	1	1	-	3	1	-	-	-	-	7
Metabolic disease	1	9	6	2	-	2	-	1	1	-	-	22
Musculoskeletal	-	1	-	6	4	5	2	1	2	-	-	21
Mental health	1	-	1	1	1	2	-	-	-	-	1	7
Multiple morbidities	1	1	3	3	3	5	1	-	1	-	-	18
Not specified	1	-	-	2	2	3	-	2	-	-	-	10
Medications Ø	-	3.9 (1–10)	2.8 (1–7)	3.6 (1–7)	7 (1–15)	5 (1–12)	1.5 (1–2)	2 (1–3)	2	-	-	
≤5	-	7	4	5	2	5	2	3	1	-	-	29
>5	-	2	1	1	3	2	-	-	-	-	-	9
Not specified	5	-	-	3	2	2	1	3	-	1	1	18

### Workshop and group experience

#### Findings from survey data

In general, the participants were absolutely satisfied with the CDSMP program (Table 4). Nearly nine out of ten (*n* =216/250, 86.7 %) reported having really enjoyed the workshop. They felt comfortable in the group, were satisfied with the course, considered its information and techniques both trustworthy and useful in everyday life, and stated that they would recommend the workshop to others. Participants also reported that they had learned how to set achievable goals and that the workshop fulfilled their expectations. Most felt that their life had improved because they had attended the program. Satisfaction with workshop organisation was extremely high, particularly relating to the collective morale, accessibility of workshop location, and comprehensive content. Participants were also extremely satisfied with the group leaders’ performance, and noted that they were well-suited for the task managing difficult situations during the workshop.

**Table 4:** Workshop experience and organisation

	M	(SD)
Satisfaction with workshop, content, activities and group situation (rated on 7-point level of agreement scale, from −3 to +3), *n* = 240-243		
Feeling comfortable in group	2.55	(0.92)
Trustworthiness of content	2.50	(0.89)
Recommending workshop to others	2.47	(1.17
Attendance was worth the effort	2.40	(1.21)
Overall satisfaction with workshop	2.22	(1.24)
Usefulness of techniques in everyday life	2.22	(1.09)
Learned to set achievable goals	2.18	(1.20)
Workshop met expectations	1.78	(1.43)
Achieved goals set before workshop	1.79	(1.24)
Workshop has significantly improved own life	1.43	(1.51)
Rating of workshop organisation and leader performance (rated on 11-point scales between 0 (low satisfaction) and 10 (total satisfaction), *n* = 234-244		
Accessibility of workshop premises and toilets	9.70	(0.88)
Workshop management of leaders	9.59	(1.09)
Comprehensibility of workshop content	9.50	(1.18)
Group atmosphere	9.41	(1.14)
Suitability of premises	9.41	(1.27)
Suitability of leaders for the task	9.37	(1.34)
Accessibility of workshop location	9.35	(1.52)
Reaction of leaders to questions and feedback	9.29	(1.37)
Leader’s management of difficult situations during workshop	9.25	(1.46)
Leader’s encouragement of participants to exchange experiences	9.12	(1.67)

Measured at the end of the workshop (only wave 2)

In terms of program feedback, the majority thought that the number of sessions, their duration, and their sequence were just right yet about one third would have preferred additional sessions (see Table 5). Both the German and French versions of the reference book were considered useful and understandable. About half (122/249; 49.0 %) of the participants had read at least half of the reference book. Focus group discussions revealed that a majority of participants (36/58; 62.1 %) continued reading the book four to six months after the workshop.

**Table 5:** Participant perspectives on potential structural and organisational changes

Item			
Number of	Just right	Preferred less	Preferred more
Participants (*n* = 241) (%)	88.0	6.6	5.4
Sessions (*n* = 242) (%)	67.6	3.7	28.6
Duration of sessions	Just right	Preferred shorter ones	Preferred longer ones
(*n* = 242) (%)	85.5	7.0	7.4
Frequency of sessions	Just right	Preferred more frequent	Preferred less frequent
(*n* = 241) (%)	91.3	3.7	5.0

Measured at the end of the workshop (only wave 2)

#### Subjective evaluation–focus groups and interviews

In the focus groups (FG) and interviews (Int), some participants had mixed feelings about the structured approach of the CDSMP. While the structure was appreciated, it was also perceived as a “tight corset”, and that its systematic process guided by the manual effected exchanges between participants and the group dynamic. Some noted frustration with time constraints and emerging discussions had to be postponed until a rest break, leading to a general wish for more discussion time and longer breaks, as well as to feelings of being restricted by time constraints (quotes below).

“The method is okay–but using the same structure six times in a row seems school-like and strict to me”. (FG2)

“Sometimes it hurts me a little bit to see that some people are directly cut off if they want to add something or that they are put off until the break”. (FG5)

Some focus groups were criticized because participants expressed a desire for additional disease-specific information. On the other hand, the simplicity and practical aspects of self-management techniques received praise. For instance, the use of ‘I-messages’ to improve patient-provider communication was experienced as powerful and highly relevant to better self-manage chronic conditions. A key thematic element was the transition towards acknowledging the challenges of living with chronic conditions as part of everyday life. Participants appreciated the comprehensive toolbox of self-management methods and the enthusiasm for the action plan that was the centrepiece of the program helped motivate them to set achievable goals (quotes below).

“I totally loved the action plan, because we did it every time, every time! We have totally internalized it”. (FG6)

“Everybody shared if something did not play out in their action plan and it was somehow good to hear that others are admitting this”. (Int2)

“What I particularly liked was the symptom cycle, the interdependence associated with the disease. I was not aware of that and was under the impression that ‘You may not react so angry all the time!’ And in the workshop, it was said that it was okay to be angry. I was so relieved to learn that it is part of the process and now I can work on dealing with it […]”. (FG7)

The value of the group experience and peer-led approach was also voiced and the group leaders living with chronic conditions seemed to have a catalyzing effect on participants. They felt accepted and overcame feelings of isolation as part of the group process:

“There were the most diverse people coming to the workshop […] and, still, there was equivalence in the group, also amongst the leaders. […] Everybody had some space”. (FG7)

“This group process and the cordiality of the people were unique. That was such a motivator being in a group and participating. This was so positive”. (FG4)

“We were ambitious doing the action plan and wanted to do it well. When we came back we wanted to be able to say: ‘At least we have tried…’–even if we did not always succeed. […] But because it was a group, right, if we are on our own we ease up”. (FG6)

Peer leaders were considered a unifying element bringing a wealth of experience that motivated participants and helped them set goals and develop individual self-management strategies. This was also evident in the workshop for female immigrants. They reported that it was a highly valuable experience, even though it was sometimes challenging given their limited language skills. Overall, these women were very positive about the variety of topics covered by the CDSMP, considered the action plan a powerful tool. Further they noted that only slight adjustments would be needed to better tailor the program.

### Self-rated changes and trends associated with the CDSMP

#### Findings from survey data

Participants exhibited improvements in perceived self-efficacy (Table 6) immediately after the workshop. They were more confident managing fatigue and doing the things they wanted to do despite pain and illness-related challenges. Initially, higher scores were evident across all items and then showed a slight decrease at follow-up (4–6 months).

**Table 6:** Perceived self-efficacy and changes in self-management of chronic condition

	(1) Start of workshop	(2) End of workshop	(3) Four to six months afterwards	Difference (*1*) *and* (*2*)
	M (SD)	M (SD)	M (SD)	(1) and (3)
Perceived self-efficacy (measured with SES6G [51]; original English text used for publication)				
How confident are you that you can…	(*n* = 228–230)	(*n* = 208–211)	(*n* = 117)	
… keep the fatigue caused by your disease from interfering with the things you want to do?	5.85	6.62	6.47	*0.77*
	(2.63)	(2.41)	(2.64)	0.62
… keep the physical discomfort or pain of your disease from interfering with the things you want to do?	5.64	6.38	6.15	*0.74*
	(2.66)	(2.38)	(2.73)	0.51
… keep the emotional distress caused by your disease from interfering with the things you want to do?	6.03	6.48	6.63	*0.45*
	(2.68)	(2.56)	(2.64)	0.60
… keep any other symptoms or health problems you have from interfering with the things you want to do?	5.93	6.64	6.26	*0.71*
	(2.50)	(2.43)	(2.64)	0.33
… do the different tasks and activities needed to manage your health condition so as to reduce your need to see a doctor?	6.73	7.24	7.01	*0.51*
	(2.63)	(2.36)	(2.76)	0.28
… do things other than just taking medication to reduce how much your illness affects your everyday life?	6.86	7.31	7.29	*0.45*
	(2.65)	(2.34)	(2.59)	0.43
Arithmetic mean and mean difference of all six scales	6.18	6.77	6.64	*0.59*
	(2.28)	(2.06)	(2.39)	0.46
Self-rated changes in self-management of chronic condition and life				
(rated on 7-point level of agreement scale, from −3 to +3)	(*n* = 228–235)	(*n* = 205–207)	(*n* = 114–117)	
Coping well with given situation in everyday life	1.62	1.86	1.99	*0.24*
	(1.80)	(1.66)	(1.60)	0.37
Handling problems related to chronic condition by oneself	1.16	1.50	1.46	*0.34*
	(1.94)	(1.57)	(1.41)	0.30
Coping with feeling down or sad at times	0.93	1.05	1.34	*0.12*
	(2.15)	(2.19)	(1.98)	0.41
Not becoming overwhelmed because of health-related difficult emotions	0.98	1.33	1.71	*0.35*
	(2.14)	(1.83)	(2.32)	0.73
Being excluded from activities in daily living because of pain^a^	−0.01	0.08	−0.08	−*0.09*
	(2.84)	(2.89)	(2.67)	0.07
Being confident to still achieve something in life	1.52	1.88	1.66	*0.36*
	(1.79)	(1.62)	(1.70)	0.14
Being positive about life despite chronic condition	1.42	1.65	1.75	*0.23*
	(1.72)	(1.72)	(1.65)	0.33
Caring about oneself	1.35	1.48	1.57	*0.13*
	(2.10)	(1.71)	(1.72)	0.22
Succeeding in setting goals and achieving them	1.26	1.62	1.63	*0.36*
	(1.81)	(1.45)	(1.77)	0.37
Paying attention about daily exercise	1.37	1.70	1.75	*0.33*
	(1.86)	(1.38)	(1.87)	0.38
Easy to relax in everyday life	0.52	0.84	0.91	*0.32*
	(2.45)	(2.08)	(1.90)	0.39
Mean difference				*0.21*
				*0.36*
Self-rated health and quality of life				
rated on 10-point scales between 0 (not occurred) and 10 (extremely severe)	(*n* = 197–229)	(*n* = 205–211)	(*n* = 111–116)	
Fatigue, exhaustion	5.70	5.06	5.04	−*0.64*
	(2.81)	(3.03)	(2.74)	−0.66
Pain	5.48	5.10	4.91	−*0.38*
	(3.02)	(3.04)	(2.76)	−0.57
Limited mobility	4.93	4.26	4.64	−*0.67*
	(3.32)	(3.31)	(3.22)	−0.29
Drowsiness, feeling down	4.78	3.09	4.36	−*0.69*
	(3.01)	(2.96)	(2.93)	−0.42
Poor concentration	4.38	3.57	3.84	−*0.81*
	(2.92)	(2.80)	(2.84)	−0.54
Insomnia	3.78	3.30	3.13	−*0.48*
	(3.42)	3.24)	(3.02)	−0.65
Discomfort	3.05	2.70	2.86	−*0.35*
	(3.19)	(3.01)	(3.11)	−0.19
Anxiety states	2.68	2.11	2.20	−*0.57*
	(3.14)	(2.83)	(2.65)	−0.48
Shortness of breath	2.29	1.19	2.15	−*0.38*
	(2.92)	(2.63)	(2.99)	−0.14
Dizziness	2.02	1.80	1.74	−*0.22*
	(2.86)	(2.84)	(2.67)	−*0.28*
Constipation	1.90	1.76	1.70	−*0.14*
	(2.86)	(2.79)	(2.87)	−0.20
Nausea	1.69	1.49	1.37	−*0.20*
	(2.53)	(3.31)	(2.24)	−0.32
Bad taste in mouth	1.70	2.00	1.32	+*0.30*
	(2.71)	(2.84)	(2.53)	−0.38
Loss of appetite	1.18	1.27	1.42	+*0.09*
	(2.23)	(2.25)	(2.29)	−0.02
Mean difference				−*0.40*
				−0.37
Self-rated changes in interaction with physician and adherence				
(rated on 7-point level of agreement scale, from −3 to +3)	(*n* = 228–235)	(*n* = 206–211)	(*n* = 114–118)	
Consider topics to be discussed with physician before consultation	2.34	2.43	2.56	*0.00*
	(1.54)	(1.28)	(0.81)	0.13
Being certain about decisions when to take medication	2.35	2.48	2.26	*0.13*
	(1.86)	(2.00)	(1.39)	−0.09
Adhering to medication regimen as prescribed	1.94	2.22	2.08	*0.28*
	(1.67)	(1.38)	(1.42)	0.14
Uncertainty and confusion after reading about new treatment opportunities^a^	−0.80	−1.21	−0.98	*0.41* ^a^
	(2.82)	(2.82)	(2.94)	0.18^a^
Mean difference				*0.06*
				−0.03
Self-rated changes in dietary behaviour				
(rated on 7-point level of agreement scale, from −3 to +3)	(*n* = 265–272)	(*n* = 241–245)	(*n* = 136–138)	
Having breakfast everyday	1.80	1.99	2.13	*0.19*
	(2.01)	(1.86)	(1.68)	0.33
Moderate use of oil and fat	1.87	2.00	1.95	*0.13*
	(1.36)	(1.15)	(1.22)	0.08
Eating a variety of foods	1.90	2.08	2.08	*0.18*
	(1.35)	(1.08)	(1.01)	0.18
Restricting use of salt	1.13	1.42	1.30	*0.29*
	(1.66)	(1.56)	(1.71)	0.05
Adequate fluid intake	1.69	1.95	1.92	*0.36*
	(1.57)	(1.40)	(1.39)	0.33
Corn or potatoes with every main meal	1.13	1.67	1.62	*0.54*
	(1.80)	(1.46)	(1.48)	0.49
Eating about the same serving with each meal	0.94	1.21	1.43	*0.27*
	(1.68)	(1.60)	(1.54)	0.49
Use of whole-grain products	0.97	1.10	1.22	*0.13*
	(1.76)	(1.69)	(1.70)	0.25
Similar meal times each day	0.87	1.22	1.21	*0.35*
	(1.72)	(1.62)	(1.48)	0.34
Sweets and snacks only occasionally	0.33	0.44	0.49	*0.11*
	(1.84)	(1.84)	(1.81)	0.16
Eating about five servings of fruit and vegetables per day	0.19	0.73	0.94	*0.54*
	(1.95)	(1.83)	(1.74)	0.75
Mean difference				*0.28*
				0.31

^a^ = negative phrased item; positive mean value indicate negative condition, difference values have inversed sign: positive difference values indicate a positive trend

After the workshop, participants reported stronger feelings of not being overwhelmed by difficult emotions triggered by their disease, they felt able to handle problems arising from their condition, and generally coping well. At follow-up (4–6 months later), they reported a slightly diminished capacity to avoid becoming overwhelmed and, their confidence in being able to handle problems. Participants also stated that compared to the start of the workshop, they felt more capable of handling feeling down/depressed at times. Agreement with various statements indicating a resolve to lead an active life also increased initially, with some showing a slight decline after 4 to 6 months (achieving something in life); however, most of these statements were rated more positively than before, and this even continued to improve over the duration of follow-up.

Most health complaints had improved by the end of the course. Participants reported fewer difficulties with concentration, less limited mobility, less fatigue, less fear, and less lack of motivation at the end of the workshop, with the largest residual improvements noted months later being fewer sleeping problems, less fear, and suffering less from dry mouth.

Interestingly, participants took more prescription medication at the end of the workshop compared to the beginning, even more than that amount four to six months later. The number of medical consultations also increased, as did nights spend in the hospital. In contrast, self-medication decreased. A change in behaviour toward one’s physician noted following the workshop, but not for the better. Participants were less likely to agree that their physician knows which medications they take; however, respondents reported generally adhering to the prescribed medication schedule.

As for diet and exercise, at workshop completion, participants reported sticking to well-known rules for healthy eating more strictly than before; and, along with the improved well-being of participants, these better eating habits persisted for several months.

#### Self-reported changes–focus groups and interviews

When asked about changes related to CDSMP attendance, participants emphasized that the program’s peer-led approach supported them developing a broader perspective regarding their condition. In the group, they were able to open up to others and talk more freely about their challenges and difficult emotions. The mentoring provided by workshop leaders and group members alike helped them to develop a more positive attitude towards their illness, and to become more hopeful and self-confident. It enabled them to better assess their own scope of action, and to become aware of the potential and limits of their self-management activities. Notably, while some regarded themselves to be active self-managers, the program provided an impetus to become re-engaged in self-care, to set priorities and to develop/adapt new self-management strategies. This was described as being encouraged to move beyond «knowing what to do» to «acting on it» by implementing self-management tools in daily life. The program provided some with a ‘Eureka moment’ as shown in the following quotes.

“It was very good for me to meet other people who have diabetes like me, but have lived with it for 43 years now–and you can’t see it. This has given me a certain level of reassurance, because I recognize that this is something you have to accept”. (FG2)

“In a way, I received confirmation that I have already done quite a lot and have always been active [when it comes to health]. But there are always new or different ways you haven’t tried yet. For me, it was reassuring that I am on track”. (FG6)

“You simply try something to find out whether it works for you or not. Where can you start to get something apparently impossible done? And then to experience this Eureka moment, the self-efficacy…” (FG7)

Participants also reported that they were now preparing for their visits with healthcare providers, and were working to develop a more trustful relationship with them, and were motivated to advocate for themselves. This transformation was also noted by family and friends who recognized considerable behavioural changes. In particular, findings from the workshop with migrant women revealed an appreciation of the opportunity to gain valuable insights into cultural practices in their host country.

## Discussion

The Stanford program has been effective in many settings. To our knowledge, this is the first systematic implementation and evaluation of the Stanford self-management program in Switzerland or any other German-speaking country, and includes preliminary insights into the early adoption of the program into a European francophone population. From the participants’ perspective, the systematic adaptation process and implementation were considered successful, resulting in a ready-for-use version of the program that can be implemented in diverse healthcare and community settings. Participants accepted the program and considered its content and methods useful, even though the structured approach does not address all the needs and expectations of people living with chronic conditions. Some short-but also long-term benefits were reported and self-efficacy improved during the small-group workshops, albeit with some slight decline in benefits several months after the workshops were over. Overall, the CDSMP had multiple positive impacts on how participants managed their chronic condition (s) and thus, is an asset for chronic illness support. Because of its peer-led approach and the simplified health information given, the program may also be relevant for vulnerable and disadvantaged populations with low health literacy.

### Process, content and methods

This study reaffirms findings from previous reports of participants’ satisfaction with a culturally-adapted version of the CDSMP [7, 10, 29, 47]. Despite a large evidence base supporting the CDSMP, it is somehow surprising that only a few details have thus far been published about how to systematically adapt the program to target specific populations or diseases [44, 45]. The three-step process (Fig. 1) can be considered a useful if not elaborate participatory model for cross-border adaption and open innovation. Patients, significant others, scholars and health professionals were involved in the adaptation process, and in constructing both structures and procedures for program implementation, which is consistent with the principles of co-creating healthcare innovations via patient engagement and reciprocal relationships to foster a co-learning process [54].

The overall highly-positive response of participants and their satisfaction with the workshops delivered in diverse settings in health-and social care are encouraging. These findings suggest that program is highly transferable. This is underscored by the coherence in focus groups discussions across settings and highlights the value of tailoring the adapted program to cultural/linguistic settings (step 2) as well as modifying the reference book for cultural effectiveness.

Overall, this suggests that the cross-border adaptation was successful. Moreover, the adapted CDSMP version works for Switzerland and other German-speaking countries. The preliminary findings from the workshops in the French-speaking region of Switzerland are also promising. Despite the usual challenges of recruiting participants, this language-specific version might lead to similar outcomes, if targeted on a large scale to European francophone’s. Indeed, previous analyses have demonstrated that translating and adapting the Stanford model results in no significant loss of effectiveness [6]. However, further analyses are needed to confirm the usefulness of the CDSMP for particular subgroups, e.g. immigrants.

Together, our survey and focus groups revealed high levels of satisfaction and the delivered health information was deemed extremely helpful. The program does not work universally for everyone. Some may consider it too long, overly restrictive, or not in line with their expectations [10, 27]. The analysis of the single workshop for people living with HIV/AIDS underscores the importance of specifying which groups will benefit most from attending a generic CDSMP workshop. This is even more important, since not being disease-specific may be considered both a strength and challenge of this generic self-management program [55]. This may help to explain why derivatives are required to target specific diseases and subgroups. Nevertheless, the relative ease of use and low-tech approach make this an appealing program for socio-economically disadvantaged people or groups with low health literacy [44, 56]. Here, an asset of the CDSMP could be that certain topics, such as action planning or exercise, are explicitly and repeatedly addressed within the workshops.

The effectiveness of specific components of the CDSMP are the subject of ongoing investigation [5, 6, 39]. Yet, our qualitative findings revealed that participants voiced a variety of supportive elements of the program which could be the focus of further investigation and innovation. It is reassuring that the action plan was viewed as a key element of the program. While some participants had to get used to the idea of setting specific goals and planning concrete actions, most individuals enjoyed using the action plans. They considered this self-management tool of high value, not only during the workshops but also in everyday life. Reports from partner organisations include examples that people were still using action plans months to more than a year after attending their workshop. These experiences reflect expectations that the action plan might be a specific component of the CDSMP enhancing self-efficacy [6]. Recent evidence supports this assumption, suggesting that the action-plan process contributes to improved self-efficacy [57]. The qualitative findings in our study identify difficulties related to the structured program, which could potentially be considered a barrier for dissemination to vulnerable groups with limited health literacy. Yet, feedback provided by female immigrants in the present study suggest otherwise. Therefore, future research activities on self-management support should also focus on the needs of vulnerable populations.

Overall satisfaction with the program is also supported by the participants’ feedback that they would have preferred additional sessions, as well as the option to continue with the program. Personal reports from program coordinators indicate that at least in some settings, workshop groups continued to meet on their own months or even years after attending the workshop. Further, they continued to work on particular action plans, in line with prior reports [56]. This suggests a need to closely link or even integrate the program into existing healthcare and social services, so as to guarantee some sort of continuity and work towards sustainable self-management support. However, as noted by the Stanford creator, while organizations can facilitate continued follow-up or reinforcement, it is up to the participants to continue activities for ongoing long term self-management following program participation.

Notably, emerging technologies could be also considered. Indeed, additional benefits have been reported with the addition of online-tools to the self-management support program (i.e. providing emotional support, enhanced peer support via exchanges of experiences, and access for hard-to-reach groups like housebound older adults) [29, 56, 58]. Another future direction could be to investigate additional delivery modes for disseminating the CDSMP in Switzerland and other German-speaking countries. Besides face-to-face interactions, this could include traditional mail or online components with social media elements targeting additional user groups and outreach rural/isolated and underserved populations [59–61]. The latter could be useful to offer additional access to emotional support that has been identified as a key theme in online self-management interventions [62]. However, given the variety of social media platforms and that this is a rapidly evolving area, using technology to enhanced pathways of future self-management support must be carefully assessed keeping in mind patients’ needs and values so that technologies that are useful for people living with chronic conditions.

### Impact and changes

The benefits (and limitations) of the CDSMP for improving self-efficacy, symptom management, health status, health-related behaviors, and relationships with healthcare providers have been extensively reported [33, 42, 63]. Due to the feasibility factor and absence of any control group in this study, our findings should be considered with caution. Some studies have shown little or no effect on self-efficacy [10, 17], our findings are reasonably consistent with evidence that it has the potential to improve it [5, 16]. In this study, self-efficacy was only considered as a mediator rather than an outcome, and further analyses are needed to investigate its sustainability, as the initially-observed improvements were followed by a slight waning in at 4–6 months. Besides the attrition rate observed in wave 3, a possible explanation for this effect might be that the program had raised expectations among participants, which could not then be fully met in the workshops [27]. Still, according to our data, the CDSMP’s effects on self-efficacy are encouraging. They suggest that elements targeting behaviour change are working; as such, it can be considered a useful supplement to chronic illness care and a worthwhile intervention when integrated into standard healthcare and social services. Moreover, it may have a positive impact upon coping resources, especially when targeting disadvantaged groups with low health literacy who have the most to gain from efforts to improve their self-management skills [6, 44].

Another important trend we observed is that participants more frequently asked for help and seemed to tome towards a team-building and collaborative approach to care. One of the CDSMP’s major strengths might be its ability to encourage respectively empower people to team up with healthcare providers (and significant others) and to master challenges related to their condition. This is supported by reports from coordinators that included examples of socio-economically disadvantaged individuals who felt particularly empowered by the group process and identified strategies to better communicate and collaborate with healthcare professionals. Past critiques of the program have included potential social comparisons related to group dynamics, which could lead to further inequalities among already-vulnerable populations [25, 39]. Along with the predominantly positive feedback provided by participants, there were no indications of such problems during the implementation process. Yet, given the limited sample size and resources of our study, further analysis is needed to more clearly define other influencing factors and effects.

One should also bear in mind that current approaches to measure the outcomes of chronic disease self-management programs might be sufficiently sensitive to identify all of the benefits of these interventions [63]. A larger perspective should be included into the, thus far, primarily RCT-driven evaluation of the CDSMP. To date this approach has predominantly focussed on clinical outcomes, yet given that some of the positive changes may not be easily-measured outcomes. Thus, future research should consider addressing the social impact of peer-led self-management support to a greater extent [64], with an emphasis on its role and relevance throughout the chronic illness trajectory. In terms of the CDSMP, this could include further analyses of underlying conceptual elements like chronic illness work [38], as these have been rarely addressed in the literature, which is predominantly driven by the logic of intervention.

### Peer-to-peer health care

A major finding of this study is the importance of role modelling by workshop leaders who themselves have a chronic illness. Participant feedback indicates that peers had an important positive effect on self-management and coping in line with previous work on peer-led self-management support [6, 7, 65]. Data from the present study and other highlight the ability of the CDSMP in aiding people in overcoming social isolation and feel supported by others [27, 28]. In addition, personal feedback from program coordinators and workshop leaders included stories of socially-isolated participants who, as a result of the action planning and role modelling, became engaged in the group process, established social connectedness. Indeed, we observed several examples of patients who initiated volunteer work in their community yielding asocial return of investment [26]. Thus the CDSMP approach can be considered a powerful example of ‘peer-to-peer healthcare’ [66] and of structured involvement of ‘experts by experience’ [67].

We considered the Stanford program as a supplement an additional tool for supporting self-management in chronic illness care. Learning from the UK debate about the ‘expert patient’ being viewed as a challenge or even a threat to health professionals, the overall message of the German and French CDSMP versions focussed less on the ideal type of patient [39] than on support from peers to deal with chronic conditions and health topics in everyday life. Thus we considered it an additional supplemental tool for supporting self-management in chronic care illness.

### Settings and implementation

While many healthcare providers are very positive regarding the peer-led standardized Stanford approach, others have been sceptical and critical of the program. This might be related to the notion that engaging peers as leaders challenges the professional identity of some providers [34, 68]. However, even though there we experienced some scepticism, providers seemed highly committed to the program as evidenced by the participants’ overall positive feedback on the quality of workshop organisation and delivery. This could be related to the standardized program [55], but it also supports the value of using a participatory process and engaging healthcare professionals during implementation.

Importantly, recruiting participants was challenging and required extra resources, findings consistent with prior reports [33, 68]. Thus, inadequate infrastructures and few participants are significant barriers for implementing the CDSMP [69]. In the present study, home care and community service organizations were more successful in recruiting patients as they had direct access to people living with chronic conditions and were delivering additional chronic illness care services. This reinforces the importance of existing infrastructures as a key element of implementation.

Linkages with such organizations might not only support ongoing engagement with healthcare professionals [70], but may also facilitate communication and allow permeability between various self-management support programs, potentially generating more coordinated care [71, 72]. To further sustain this and to strengthen social inclusion, post-workshop follow-up may be needed. We received positive feedback from organizations delivering the workshops pertaining to the relevance of such a continuous, ongoing group-based approach, which was requested by a number of participants. In fact, in the absence of a continuing program some participants voluntarily opted to attend workshops twice. Some participating organizations addressed this need by establishing regular informal meeting opportunities (‘Evivo Café’) to facilitate continued social interactions.

The analysis of the immigrant group suggests that tailored approaches to specific patient groups may be effective. People with mental health problems are of particular relevance for future CDSMP work as such programs have been previously shown to be effective in such populations [20, 44]. These participants were well-represented in our sample (21.4 %), even though no mental health service provider was directly involved in piloting the program. Thus, offering small group workshops to people living with mental health problems might be an effective strategy for these patients. Indeed, a similar approach has been used to reach those who are unemployed and who may benefit from self-management programs supporting motivation and behavioural change^3^

### Study strengths and limitations

Like all studies of this kind, this project has several limitations. First, we relied heavily on self-reported outcome measures, the evaluation was conducted without a comparison group, lacked randomization and thus our study findings must be interpreted with care. Second, the follow-up period was relatively short and we had attrition during the four to six months interim. Therefore, it remains an open question how certain outcomes, like self-efficacy, might have been affected if more participants completed the final survey. We did not have sufficient follow-up on those who dropped out to determine if the reason for drop-out was related to their health, the workshop itself, or the length of the questionnaire. Last, are issues related to the sample which are similar to other CDSMP-related studies [47] and likely characteristic of research on self-management support in chronic illness itself, as all those who ‘opted in’ were, by definition, motivated volunteers. They also generally were well-educated, some were probably already good self-managers, and over 90 % were women. All these factors have been cited as criticisms in other studies, wherein the self-selection process resulted in the failure to recruit socioeconomically-deprived individuals [37, 69, 70].

## Conclusions

Our findings emphasize the value of peer-led self-management support as a key element of chronic illness care, and as an asset for improving health literacy and the empowerment of patients and their significant others. The adapted CDSMP (Evivo) was successfully transferred into the cultural context of Switzerland and Austria and can be expected to also work in other German-and French-speaking European countries.

Despite a variety of patient-education activities, peer-led self-management support remains in its infancy in Austria, Germany and Switzerland [73, 74]. As health policy awareness increases regarding the need to empower patients and foster health literacy [75], the role of peers in patient education and self-management support is growing. Given the complexity of the Stanford program itself and the challenges that delivering organisations experienced (e.g., with recruitment), the program should be reasonably integrated and linked into existing health and social care services, so it can yield its benefits as a valuable supplement instead of being used as a stand-alone solution. This ensures better access for participants and enhances sustainability. If broadly implemented, the adapted CDSMP could have meaningful, wide-ranging and complementary implications for chronic illness care, as well as for the primary and tertiary prevention of chronic disease [42]. Here, new technologies might offer additional ways of dissemination to make the program more accessible to hard-to-reach or vulnerable individuals who have the most to gain [44, 56]. Finally, there is a need to further investigate, amongst other issues, the cost effectiveness of the adapted CDSMP, its social impact and returns on investment, and whether disease-specific derivatives of the program might be of value for chronic illness care, particularly within French- and German-speaking regions of Europe.

## Footnotes

1 «Evivo» is the brand used in Switzerland along with the Stanford workshop that is labeled «Gesund und aktiv leben» (English translation: «Living healthy and actively»). Evivo symbolizes empowerment (“e”) as well as living with chronic conditions (derived from Latin, “vivo” meaning “I live”) in one word. It has been developed so the program has an empowerment notion and its name can be used in four national languages in Switzerland (German, French, Italian and also Rhaeto-Romanic). Organisations running the adapted Stanford program are embedded in the non-profit association «Evivo Network», which was founded in 2014 to promote patient empowerment and the sustainable future implementation of the Stanford model in Switzerland and German-speaking Europe. For more information: www.evivo.ch (visited December 2015).

2 Since 2015, there have been new efforts to deliver CDSMP workshops in Germany, which are not part of the study at-hand. The program «Living healthy and actively» is disseminated by the multi-stakeholder consortium INSEA (accronym stands for «Initiative für Selbstmanagement/Selbsthilfe, Empowerment und Aktiv Leben»; English Translation: «Initiative for Self-Management/Self-help, Empowerment and Active Living»). It currently consists of a regional self-help organization (Selbsthilfekontaktstelle Bayern, Germany) and the first mini-med school in Germany (Patient University, Hannover), which are delivering the workshops and are supported by two non-profit foundations (Robert Bosch Foundation, Germany; Careum Foundation, Switzerland) and a health insurance fund (Barmer GEK, Germany). Unlike the unsuccsessful 1st attempt in Germany, the two organisations now delivering the workshops have sustainable field access at their disposal and additional funding at hand. For more information, visit www.insea-aktiv.de (visited December 2015)

3 See www.patientuddannelse.info (visited December 2015)
